# Circular RNA hsa_circ_0006117 Facilitates Pancreatic Cancer Progression by Regulating the miR-96-5p/KRAS/MAPK Signaling Pathway

**DOI:** 10.1155/2021/9213205

**Published:** 2021-09-02

**Authors:** Tao Liu, Lei Zhou, Zhiwei He, Yankun Chen, Xueyi Jiang, Jian Xu, Jianxin Jiang

**Affiliations:** ^1^School of Clinical Medicine, Guizhou Medical University, Guiyang 550000, China; ^2^Department of Hepatic-Biliary-Pancreatic Surgery, The Affiliated Hospital of Guizhou Medical University, Guiyang 550000, China; ^3^Department of Hepatobiliary Surgery, Renmin Hospital of Wuhan University, Wuhan 430060, China

## Abstract

Circular RNAs (circRNAs) play key roles in many malignant tumors, including pancreatic cancer (PC); however, whether circular RNA hsa_circ_0006117, a newly identified circRNA, has a role in PC has not been investigated. Here, in order to elucidate the role and potential molecular mechanisms of circRNAs, we utilized bioinformatic tolls to screen the differentially expressed circRNAs in PC. Subsequently, circular RNA hsa_circ_0006117 was identified as being highly expressed in PC tissues in a screen of two GEO datasets, which was further verified in PC cell lines and tissues. Then, its molecular characteristics were investigated using methods such as Sanger sequencing and fluorescence in situ hybridization (FISH). Functional experiments subsequently indicated that circular RNA hsa_circ_0006117 facilitated the malignant behaviors of PC cells, prompting that it plays an oncogenic role in PC. Moreover, we found that circular RNA hsa_circ_0006117 exerts its PC-promoting effects via activating the KRAS/mitogen-activated protein kinase (MAPK) signaling pathway. Through bioinformatics exploration and dual-luciferase reporter assays, miR-96-5p was identified as a downstream target of circular RNA hsa_circ_0006117. A series of assays confirmed that circular RNA hsa_circ_0006117 acted as a miR-96-5p sponge, thereby promoting the malignant features of PC in a miR-96-5p/KRAS axis-dependent manner. Taken together, our study indicated, for the first time, that the specifically highly expressed circular RNA hsa_circ_0006117 facilitates PC progression via the modulation of the miR-96-5p/KRAS/MAPK signaling pathway and might be a hopeful therapeutic target for PC.

## 1. Introduction

Pancreatic cancer (PC) is a leading cause of cancer-related deaths worldwide among all cancers with the lowest five-year overall survival rate (10%) [1]. Although surgical resection, neoadjuvant therapies, and comprehensive targeted treatment have improved the treatment options, early metastasis and invasion, combined with the lack of effective and precise targeted therapies, still limit the prognosis of PC patients [2–4]. Indeed, a severely poor prognosis with overall survival of just 10–16 months is usually related to patients with locally advanced or unresectable PC [5]. This highlights the urgency in elucidating the molecular regulatory network in PC and searching for new or more effective therapeutic targets for treating disease.

Increasing evidence suggests that noncoding RNAs have widely participated in PC development [6–8]. Circular RNAs (circRNAs) are a sort of noncoding RNA stemmed from the back-splicing of precursor messenger RNAs (mRNAs). They have a stable covalent circular structure and are resistant to digestion by RNase R [9]. Compared with their linear precursors, circRNAs have functions that are independent of their host genes [10], such as serving as microRNA (miRNA) sponges or protein scaffolds, interacting with RNA-binding proteins, regulating alternative splicing or transcription, and generating pseudogenes [11, 12]. Emerging shreds of evidence have revealed that circRNAs act as miRNA sponges to affect downstream targets and play roles in various cancers, including PC [13, 14]. Nevertheless, the roles of circRNAs in PC remain poorly understood.

Members of the rat sarcoma (RAS) oncogene family, including Kirsten-RAS (*KRAS*), Harvey-RAS (*HRAS*), and neuroblastoma-RAS (*NRAS*), exhibit the highest mutation frequency in human cancers, with associated mutations being identified in approximately 30% of all cancers [15]. *KRAS* is a driver gene of many diseases and one of the most common and frequently mutated genes in PC [16, 17]. *KRAS* oncogenic mutations lead to the continued activation of downstream molecules, and the *KRAS*/mitogen-activated protein kinase (MAPK) signaling pathway is strongly associated with the development of PC, both of which enhance the malignant potential of this cancer [18, 19]. Meanwhile, *KRAS* is also involved in the regulation of noncoding RNAs in some cancers [20–22]. However, the association between circRNAs and *KRAS* in PC has not been explored, nor has the underlying regulatory relationship.

Here, circular RNA hsa_circ_0006117 with high expression in PC was reidentified from two circRNA microarrays from the Gene Expression Omnibus (GEO) database. Subsequent functional experiments illustrated that circular RNA hsa_circ_0006117 promoted the rapid development of PC cells. Moreover, our results suggested that circular RNA hsa_circ_0006117 could activate the MAPK signaling pathway by relieving the miR-96-5p-mediated posttranscriptional suppression of *KRAS*. Taken together, we found that circular RNA hsa_circ_0006117 adsorbed miR-96-5p and acted in an axis-dependent regulation of miR-96-5p/KRAS/MAPK, thereby facilitating proliferation, migration, and invasion in PC. Circular RNA hsa_circ_0006117 has the potential to represent a promising target for PC therapy.

## 2. Materials and Methods

### 2.1. Acquisition of Gene Expression and Identification of Differentially Expressed circRNAs (DECs)

Using the keywords “circular RNA,” “pancreatic cancer” or “pancreatic ductal adenocarcinoma,” the expression profile of circular RNAs in PC were searched from the GEO (https://www.ncbi.nlm.nih.gov/geo/) database. The microarray datasets GSE69362 and GSE79634 were included and downloaded for screening potential DECs. After identifying DECs using the R “limma” package, Venn diagram analysis (http://bioinformatics.psb.ugent.be/webtools/Venn/) was performed to overlap and focus candidate DECs.

### 2.2. Tissue Specimens and Cell Culture

We collected fresh pancreatic tissue specimens from 20 patients who had undergone pancreaticoduodenectomy or distal pancreatectomy at the Affiliated Hospital of Guizhou Medical University, China. A pathological evaluation was employed to confirm the presence of PC. All patients with PC provided informed consent. This project was approved by the Ethics Committee of Guizhou Medical University (Approval Number: 2021 No. 129). Normal human pancreatic ductal epithelial cell (HPDE) was purchased from Beijing North Carolina Chuanglian Biotechnology Research Institute (Beijing, China). All PC cell lines were purchased from American Type Culture Collection (Manassas, VA, USA), including PANC-1, MIA PaCa-2, AsPC-1, BxPC-3, and SW1990. The cells were cultured in Dulbecco's modified Eagle's medium (DMEM) (Gibco, Waltham, USA) or Roswell Park Memorial Institute (RPMI) medium (Gibco) supplemented with 10% fetal bovine serum (Gibco).

### 2.3. RT-qPCR, Agarose Gel Electrophoresis, and Sanger Sequencing

We used an RNA-easy Isolation Reagent (Vazyme, Nanjing, China) to extract RNA based on its protocol. Real-time quantitative reverse transcription PCR (RT-qPCR) was performed using cDNA Synthesis Kit (Vazyme) and qPCR Probe Kit (Vazyme). Relative mRNA expression was analyzed with the 2^−ΔΔCT^ method and standardized to those of the appropriate internal references. All primers are displayed in Table 1. Divergent primers were used for PCR amplification of reverse-transcribed cDNA and 2% agarose gel was selected for agarose gel electrophoresis. Then, agarose gel was used for Sanger sequencing with the assistance of Cloud-Seq Biotech (Shanghai, China).

### 2.4. RNase R Assay and Actinomycin D Treatment

Total RNA (2 *μ*g) was incubated with or without 3 U/*μ*g RNase R (Epicentre, Madison, WI, USA) at 37°C for 30 minutes. Then, RT-qPCR was used for analyzing the abundance of circular RNA hsa_circ_0006117 and its parent gene protein tyrosine phosphatase receptor type A (PTPRA). Meanwhile, PC cells were treated with 2 *μ*g/mL actinomycin D (Sigma-Aldrich, St Louis, MO, USA) or dimethyl sulfoxide (DMSO) (Sigma-Aldrich) for 12 hours. After harvesting the cells, the stability of circular RNA hsa_circ_0006117 and linear RNA PTPRA was tested by RT-qPCR.

### 2.5. Separation of Cytoplasmic and Nuclear Fractions

All subcellular RNA components of PC cells were isolated with a Cytoplasmic & Nuclear RNA Purification Kit (Norgen Biotek, Thorold, ON, Canada) based on the protocol. While the cytoplasmic/nuclear RNA ratio was assessed by RT-qPCR, U6 was used as a positive reference for nuclear RNA and GAPDH as a positive reference for cytoplasmic RNA.

### 2.6. Fluorescence In Situ Hybridization (FISH)

A FISH Kit (RiboBio, Guangzhou, China) was applied to visualize the subcellular localization of circRNA. PC cells were sowed in 24-well plates and reproduced until 60%–70% confluence. After fixation in 4% paraformaldehyde and permeabilization with TritonX-100, the cells were hatched with a prehybridization buffer for 30 min at 37°C and then with a Cy3-labeled circular RNA hsa_circ_0006117 probe (RiboBio) in hybridization buffer overnight at 37°C. Representative pictures were obtained using a fluorescence microscope (Olympus, Tokyo, Japan) at ×400 magnification.

### 2.7. Western Blot

Dissolving PC cells with RIPA lysis buffer (Boster, Wuhan, China), 10% SDS-PAGE was used to segregate proteins and then transferred them onto PVDF membranes (Millipore, MA, USA). After blocking with 5% milk (total protein) or 5% BSA (phosphorylated protein), the membranes were hatched with the corresponding primary antibodies and an HRP-conjugated secondary antibody. Immunoreactive protein bands were exposed with an ECL reagent (Boster) and quantified by Image Lab Software. The following commercially antibodies were used in this experiment: KRAS, GAPDH, phosphorylated-MEK1/2 (P-MEK1/2), MEK1/2, phosphorylated-ERK1/2 (P-ERK1/2), and ERK (GAPDH and KRAS from ABclonal Technology, Wuhan, China; others from Cell Signaling Technology, Beverly, MA, USA).

### 2.8. Plasmids, Oligonucleotides, and Transfection

All oligonucleotides (miR-96-5p-inhibitor or mimics, small interfering RNA [siRNA]) targeting circular RNA hsa_circ_0006117, and their controls) were obtained from RiboBio (Guangzhou, China). The KRAS expression vector (pcDNA) was purchased from Vigenebio (Shandong, China). Opti-MEM and Lipofectamine 3000 (Invitrogen, Carlsbad, CA, USA) were performed to cotransfect constructs into PC cells. Then, short hairpin RNA (shRNA) targeting circular RNA hsa_circ_0006117 was generated by GeneChem (Shanghai, China).

### 2.9. Cell Counting Kit-8 (CCK-8) and Colony Formation Assay

PC cells transfected with 2000 cells/well were treated with CCK-8 reagent (CCK-8 kit; Bosterbio, China). At a specific time (6 h, 24 h, 48 h, 72 h, and 96 h), a microplate reader (Thermo Fisher Scientific, Waltham, MA, USA) was used to record the absorbance at 450 nm. Transfected PC cells were incubated with 800 cells/plate for 12 days for colony formation assay. After imaging, the number of clones was calculated and statistically analyzed.

### 2.10. Wound Healing Assay and Transwell Assay

After the transfected PC cells were planted, a 200 *μ*L pipette tip was applied to scratch the monolayer in transfected PC cells. Representative pictures were obtained with an inverted microscope (Olympus) at the appointed times (0 and 48 h). The migration rate was normalized using the 0 h scratch area. At the same time, transwell assays was used to estimate the migratory and invasive capabilities of PC cells. The upper chamber was pretreated or not with 60 *μ*L Matrigel (Matrigel BD Biosciences, NY, USA) before transfection of 200 *μ*L serum-free PC cells (5 × 10^4^ cells/well). And 600 *μ*L of 20% FBS medium was placed in the lower chamber. Migrating or invading cells were counted after 24–28 h of incubation. Representative pictures were captured with an inverted microscope (Olympus).

### 2.11. Circular RNA hsa_circ_0006117 Target Prediction and Bioinformatic Analysis

CircBANK (http://www.circbank.cn/searchCirc.html), TargetScan (http://www.targetscan.org/vert_72/), miRDB (http://mirdb.org/), and miRTarBase (http://miRTarBase.cuhk.edu.cn/) were performed to postulate circular RNA hsa_circ_0006117/miRNA binding and mRNA/miRNA binding, respectively. Meanwhile, the “clusterProfiler” package in R was used for Kyoto Encyclopedia of Genes and Genomes (KEGG) enrichment analysis to ascertain the molecular regulatory network of circular RNA hsa_circ_0006117 using a threshold of *P* value <0.05.

### 2.12. Dual-Luciferase Reporter Assay

The circRNA-miRNA and miRNA-mRNA binding sites were predicted by StarBase (http://starbase.sysu.edu.cn/) and TargetScan. The circular RNA hsa_circ_0006117 sequence and that of the 3′untranslated region (3′UTR) of KRAS were amplified and separately inserted into the pmiR-RB-Report (Ribobio), termed as circRNA-WT or KRAS-WT. Meanwhile, the corresponding mutant sequences were also amplified and inserted into the same vector to generate circRNA-MUT and KRAS-MUT, respectively. After the cotransfection of these vectors (50 ng) and miR-96-5p control or miR-96-5p mimics (50 nm) into PC cells, a GLOMAX 96 spectrophotometer (Promega) and the Dual-Glo Luciferase Assay System (Promega, Madison, WI, USA) were applied to detect luciferase activity.

### 2.13. Statistical Analysis

SPSS version 26.0 (SPSS IBM, Armonk, NY, USA) was applied for statistical analysis. Independent sample Student's *t*-tests were used for comparisons between two groups, whereas one-way ANOVA was employed for comparisons of more than two groups. Pearson's correlation curve analysis was applied for assessing the correlations between different indicators. All tests were two-tailed, and a *P* value of <0.05 suggested statistical significance.

## 3. Results

### 3.1. Identification and Characterization of Circular RNA hsa_circ_0006117

**Figure 1:**
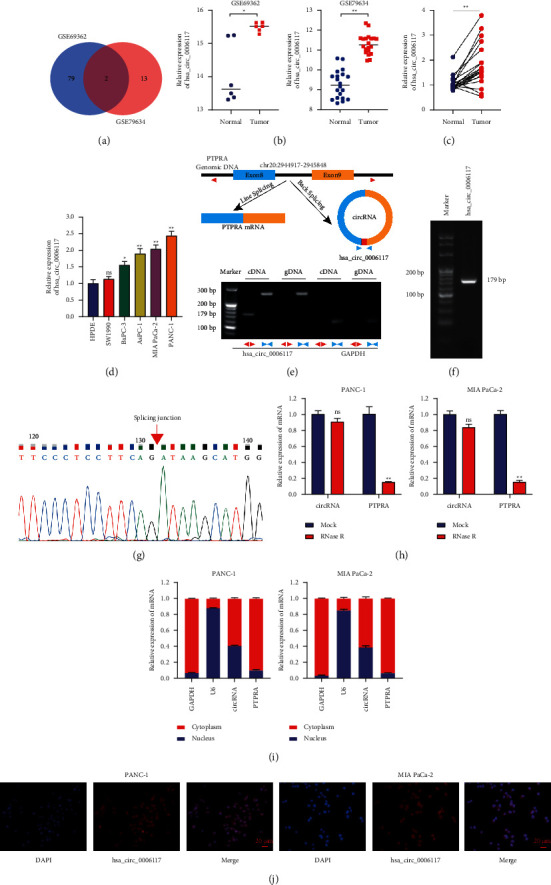
The identification and characterization of circular RNA hsa_circ_0006117. (a) Differentially expressed circRNAs that are upregulated in both GSE69362 and GSE79634 datasets. (b) Circular RNA hsa_circ_0006117 is differentially expressed between pancreatic cancer (PC) and paracancerous samples in GSE69362 and GSE79634. (c) The expression of circular RNA hsa_circ_0006117 in 20 pairs of matched PC tissues (tumor) and the corresponding paracarcinoma tissues (normal). (d) Circular RNA hsa_circ_0006117 expression in PC cell lines was tested by RT-qPCR. (e) Upper panel: schematic illustrations showing the genomic location of PTPRA and that of circular RNA hsa_circ_0006117 derived from a region 5′ of exon 8 to 3′ of exon 9 of the PTPRA locus. Lower panel: agarose gel electrophoresis analysis of circular RNA hsa_circ_0006117 and GAPDH in cDNA and genomic DNA (gDNA) derived from MIA PaCa-2 cells. (f) RT-PCR with divergent primers showed that circular RNA hsa_circ_0006117 was expressed in cDNA derived from MIA PaCa-2 cells. (g) The back-splicing junction (red arrow) associated with circular RNA hsa_circ_0006117 generation as identified by Sanger sequencing. (h) The relative mRNA expression of circular RNA hsa_circ_0006117 and PTPRA in PC cells after RNase R treatment. (i, j) The subcellular distribution of circular RNA hsa_circ_0006117 was ascertained by RT-qPCR (i) and fluorescence in situ hybridization (FISH) (j). GAPDH and U6 were used as reference indicators for the subcellular distribution of circular RNA hsa_circ_0006117 in the cytoplasm and nucleus, respectively. The circular RNA hsa_circ_0006117 probe was labeled with Cy3 (red) and the nucleus was counterstained with DAPI (blue). Representative images were obtained at ×400 magnification (bars: 20 *μ*m). All values were shown as means ± SD, ns: not significant, ^*∗*^*P* < 0.05, and ^*∗∗*^*P* < 0.001.

We identified potential DECs from GSE69362 and GSE79634. Venn diagram analysis showed that the overlapping circRNAs (circular RNA hsa_circ_0006117 & circular RNA hsa_circ_0029634) in two datasets were both elevated in PC tissues (Figure 1(a) and Supplementary figures S1A and S1B). We further found that circular RNA hsa_circ_0006117 (Figure 1(b)) was differentially expressed between PC and paracancerous samples in each data set. Then, we concentrated on exploring circular RNA hsa_circ_0006117. RT-qPCR analysis indicated that circular RNA hsa_circ_0006117 (Figure 1(c)) was significantly upregulated in 20 pairs of PC tissues. Meanwhile, circular RNA hsa_circ_0006117 expression was upregulated in PC cells compared with that in HPDE cells (Figure 1(d)). In terms of the annotation in circBase (http://www.circbase.org/), we found that circular RNA hsa_circ_0006117 is derived from 5′ of exon 8 to 3′ of exon 9 of *PTPRA* (Chr20: 2944917–2945848) by back-splicing (Figure 1(e), upper panel). Thus, we designed divergent and convergent primers to amplify the back-spliced (circular RNA hsa_circ_0006117) and linear products (*PTPRA*), respectively. Circular RNA hsa_circ_0006117 could only be amplified from cDNA obtained from MIA PaCa-2 cells using divergent primers (Figure 1(e) (lower panel), Figure 1(f)). Next, Sanger sequencing of PCR amplicons validated the back-splicing site of circular RNA hsa_circ_0006117 (Figure 1(g)). After that, the stability of circular RNA hsa_circ_0006117 in PC cells treated with RNase R or actinomycin D was analyzed. RT-qPCR results revealed that circular RNA hsa_circ_0006117 had strong tolerance to RNase R digestion, whereas the linear form of *PTPRA* was rapidly degraded (Figure 1(h)). A stability assay using actinomycin D treatment also demonstrated that circular RNA hsa_circ_0006117 was substantially more stable than linear *PTPRA* in PC cells (Supplementary figure S1C). Furthermore, separation of cytoplasmic and nuclear fractions (Figure 1(i)) and FISH (Figure 1(j)) suggested that the subcellular localization of circular RNA hsa_circ_0006117 was mainly in the cytoplasm.

### 3.2. Circular RNA hsa_circ_0006117 Facilitated the Proliferation, Migration, and Invasion of PC Cells

**Figure 2:**
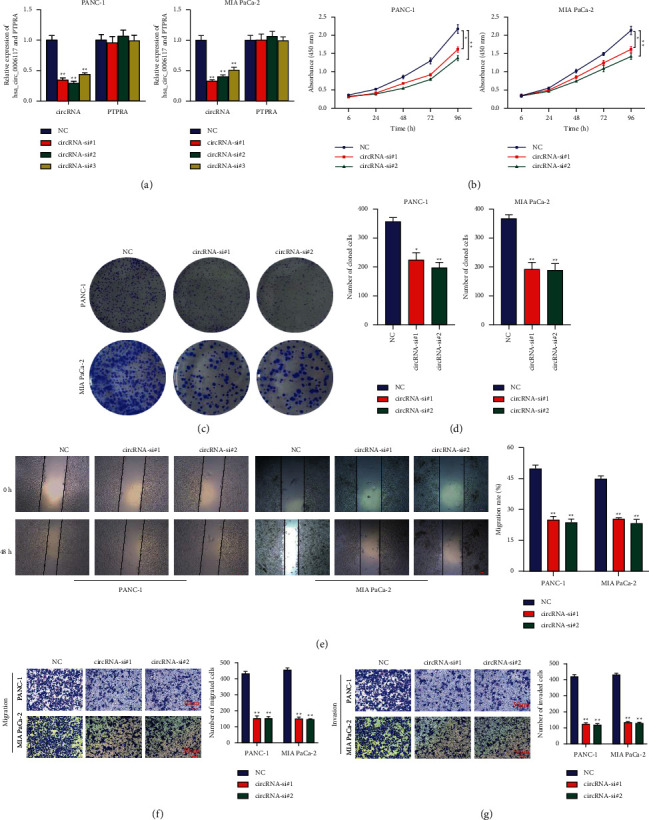
Circular RNA hsa_circ_0006117 facilitates the proliferation, migration, and invasion of pancreatic cancer (PC) cells. (a) The mRNA expression of circular RNA hsa_circ_0006117 and PTPRA in circular RNA hsa_circ_0006117-silenced PC cells. (b) Cell counting kit-8 (CCK-8) assays in circular RNA hsa_circ_0006117-silenced PC cells. (c, d) Colony formation assays in circular RNA hsa_circ_0006117-silenced PC cells. (e) Wound healing assays in circular RNA hsa_circ_0006117-silenced PC cells. Representative images were obtained at ×40 magnification (bars: 100 *μ*m). (f, g) Transwell migration (f) and invasion (g) assays in circular RNA hsa_circ_0006117-silenced PC cells were applied to evaluate the invasiveness and migration capabilities of PC, respectively. Representative images were obtained at ×200 magnification (bars: 50 *μ*m). All values were shown as means ± SD, ^*∗*^*P* < 0.05, and ^*∗∗*^*P* < 0.001.

A series of loss-of-function experiments were applied to illustrate the function of circular RNA hsa_circ_0006117 in PC. For this, we designed siRNAs targeting the unique back-splicing site of circular RNA hsa_circ_0006117 (Supplementary figure S2) and used RT-qPCR to confirm that transfection of these siRNAs reduced the expression of circular RNA hsa_circ_0006117, but not that of *PTPRA* (Figure 2(a)). Because circRNA-si#1 and circRNA-si#2 elicited the best results, they were used for subsequent experiments. The proliferation capacity was suppressed by the downregulation of circular RNA hsa_circ_0006117 in PC cells, which was confirmed by CCK-8 (Figure 2(b)) and colony formation (Figures 2(c) and 2(d)) assays. Moreover, both wound healing (Figure 2(e)) and transwell (Figures 2(f) and 2(g)) assays indicated that downregulating circular RNA hsa_circ_0006117 markedly weakened the invasiveness and migratory capability of PC cells.

### 3.3. Circular RNA hsa_circ_0006117 Maintained the Malignant Characteristics of PC via Activating the MAPK Signaling Pathway

**Figure 3:**
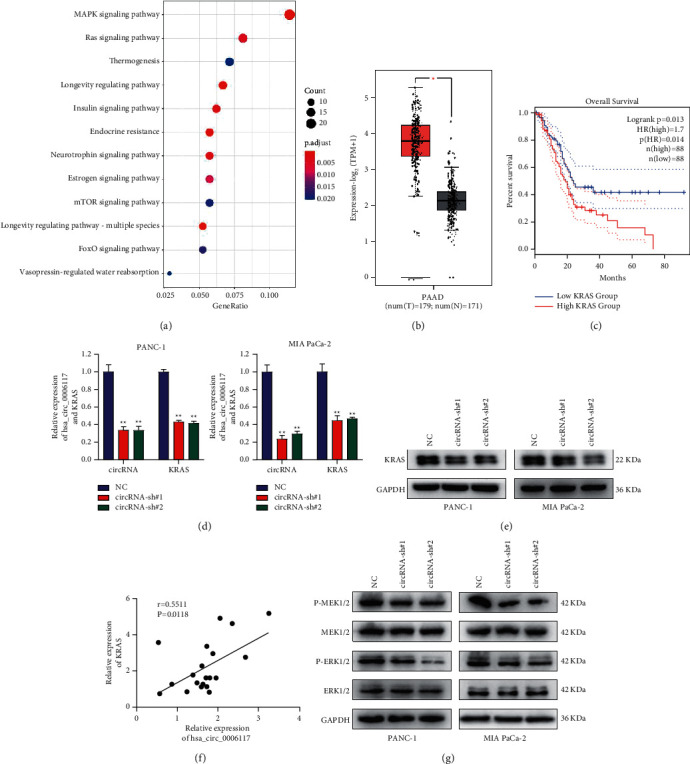
Circular RNA hsa_circ_0006117 maintains the malignant characteristics of pancreatic cancer (PC) via activating the MAPK signaling pathway. (a) The potential circular RNA hsa_circ_0006117-associated signaling pathways were identified by KEGG analysis in R. (b) *KRAS* is highly expressed in PC samples from the TCGA database, which was queried by the GEPIA database. (c) The overall survival data of PC patients obtained from the TCGA database; the dotted line indicates the 95% confidence interval. (d, e) RT-qPCR and western blot assays in circular RNA hsa_circ_0006117-silenced PC cells were adopted to measure the mRNA (d) and protein content (e) of KRAS. (f) The correlation between circular RNA hsa_circ_0006117 and KRAS was estimated by Pearson's correlation curve analysis. (g) The expression of P-MEK1/2, MEK1/2, P-ERK1/2, and ERK1/2 were detected in circular RNA hsa_circ_0006117-silenced PC cells. All values were shown as means ± SD, ^*∗*^*P* < 0.05, and ^*∗∗*^*P* < 0.001.

Studies have suggested that exon-derived circRNAs mainly exert their functions in tumor development by sponging miRNAs [12]. To explore whether circular RNA hsa_circ_0006117 functions as a miRNA sponge, we used bioinformatics databases, such as circBANK, miRDB, TargetScan, and miRTarBase, to predict the targets of circular RNA hsa_circ_0006117 within the competitive endogenous RNA network associated with this circRNA. Meanwhile, we found that the MAPK and RAS signaling pathways were the most likely downstream targets of circular RNA hsa_circ_0006117 in KEGG enrichment analysis (Figure 3(a)). Subsequently, combined with gene expression profile from The Cancer Genome Atlas (TCGA) database, we focused on genes involved in the MAPK signaling pathway and identified highly expressed *KRAS*, *GRB*2, *IGF2BP*2, and *RAP*1*A* in PC tissues as the most significantly enriched genes, implicating them as potential targets of circular RNA hsa_circ_0006117 (Figure 3(b) and Supplementary figures S3A–S3C). In addition, we found that *KRAS* reduced overall survival in patients with PC based on data from the Gene Expression Profiling Interactive Analysis (GEPIA) database (http://gepia2.cancer-pku.cn/#index) (Figure 3(c)). Therefore, we further constructed shRNAs using the sequence of circRNA-si#1 and circRNA-si#2 and then transfected them into PC cells for follow-up experiments. Interestingly, the mRNA (Figure 3(d) and Supplementary figure S3D) and protein (Figure 3(e)) expression of *KRAS* were both downregulated in PC cells transfected with shRNA targeting circular RNA hsa_circ_0006117. Moreover, a direct relationship between the expression of circular RNA hsa_circ_0006117 and *KRAS* in PC tissue samples was revealed by Pearson's correlation analysis (Figure 3(f)). It is well known that *KRAS* contributes to the upregulation of the MAPK signaling pathway, we hypothesized that circular RNA hsa_circ_0006117 was also involved in the influence on the MAPK signaling pathway. We verified that the protein levels of phosphorylated mitogen-activated extracellular signal-regulated kinase 1/2 (P-MEK1/2) and phosphorylated extracellular signal-regulated kinases 1/2 (P-ERK1/2) were lower in circular RNA hsa_circ_0006117-silenced PC cells than in the negative controls, whereas the total MEK1/2 and ERK 1/2 protein level remained unchanged (Figure 3(g)).

### 3.4. The Circular RNA hsa_circ_0006117-Mediated Malignant Progression of PC Was Dependent on KRAS

**Figure 4:**
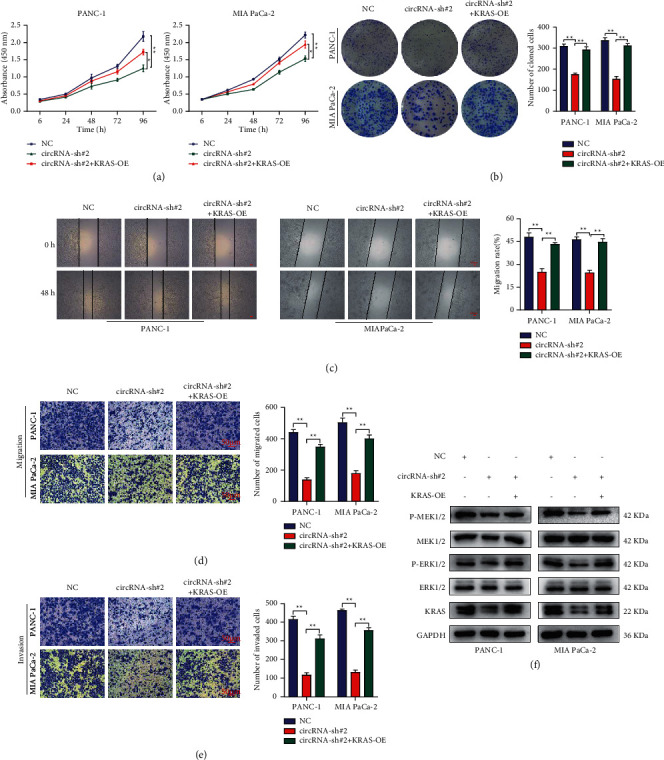
The circular RNA hsa_circ_0006117-mediated malignant progression of pancreatic cancer (PC) is *KRAS*-dependent. (a) The growth ability of PC cells was confirmed with cell counting kit-8 (CCK-8) assay in circular RNA hsa_circ_0006117-silenced and (or) *KRAS*-overexpressing PC cells. (b) The colony-forming ability of PC cells was evaluated by colony formation assay in circular RNA hsa_circ_0006117-silenced and (or) *KRAS*-overexpressing PC cells. (c) The migratory capacity of PC cells was estimated by wound healing assay in circular RNA hsa_circ_0006117-silenced and (or) *KRAS*-overexpressing PC cells. Representative images were obtained at ×40 magnification (bars: 100 *μ*m). (d, e) The invasive and migratory capabilities of PC cells in rescue experiments were investigated by transwell migration (d) and invasion (e) assays, respectively. Representative images were obtained at ×200 magnification (bars: 50 *μ*m). (f) The expression of key proteins associated with the MAPK signaling pathway in rescue experiments. All values were shown as means ± SD, ^*∗*^*P* < 0.05, and ^*∗∗*^*P* < 0.001.

To verify whether circular RNA hsa_circ_0006117 facilitates PC progression through regulating *KRAS*, we overexpressed *KRAS* in circular RNA hsa_circ_0006117-silenced PC cells to rescue the inhibitory effects of circRNA-sh#2. Proliferative capacity assays revealed that ectopically expressed *KRAS* could partially rescue the effects induced by circular RNA hsa_circ_0006117 silencing and facilitated the growth of PC cells (Figures 4(a) and 4(b)). Similarly, wound healing (Figure 4(c)) and transwell (Figures 4(d) and 4(e)) assays demonstrated that *KRAS* overexpression partially rescued the circular RNA hsa_circ_0006117 knockdown-induced effects and reinforced the migration and invasion potential of PC cells. Furthermore, in PC cells where circular RNA hsa_circ_0006117 was knocked down, *KRAS* transfection partially restored the expression of P-MEK1/2 and P-ERK1/2 when the total MEK1/2 and ERK1/2 remained constant (Figure 4(f)).

### 3.5. The Circular RNA hsa_circ_0006117-Dependent KRAS Regulation in PC Progression Was Mediated by miR-96-5p

**Figure 5:**
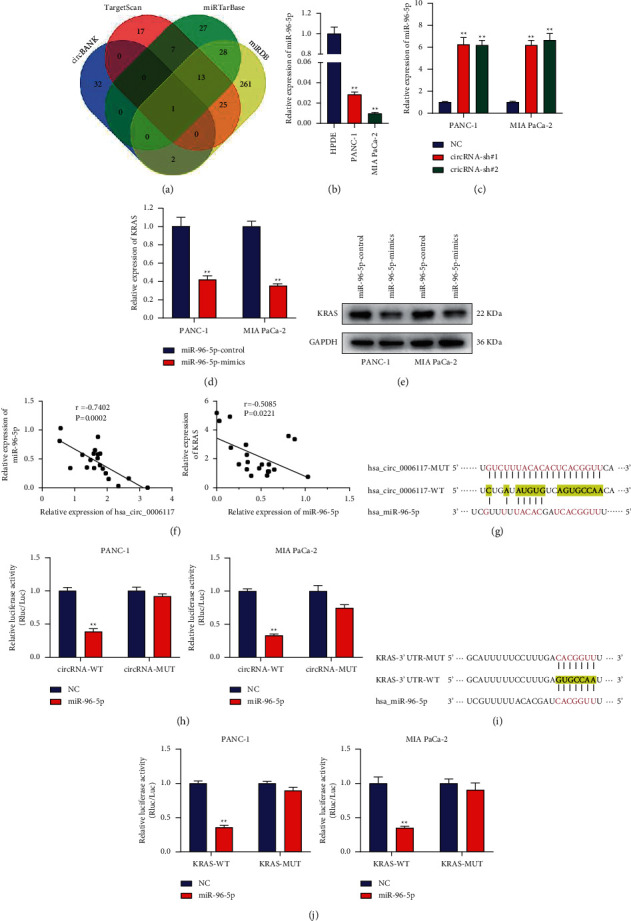
MiR-96-5p mediates the circular RNA hsa_circ_6117-associated regulation of KRAS in the progression of pancreatic cancer (PC). (a) A Venn diagram showing the overlapping miRNAs that simultaneously bind to circular RNA hsa_circ_0006117 and KRAS as predicted by circBANK, TargetScan, miRDB, and miRTarBase. (b) Compared with HPDE cells, RT-qPCR showing the mRNA content of miR-96-5p in PC cells. (c) The mRNA content of miR-96-5p was detected in circular RNA hsa_circ_0006117-silenced PC cells. (d, e) The mRNA (d) and protein content (e) of *KRAS* in miR-96-5p-overexpressing PC cells. (f) Pearson's correlation curve analysis provided a direct link between miR-96-5p and circular RNA hsa_circ_0006117 or *KRAS*. (g) A schematic diagram showing the putative conjugated site between circular RNA hsa_circ_0006117 and miR-96-5p. (h) The relative luciferase intensity in PC cells cotransfected with the miR-96-5p mimics or miR-96-5p control and circRNA-WT (containing the wild-type (WT) circular RNA hsa_circ_0006117 sequence) or circRNA-MUT (containing a mutated [MUT] circular RNA hsa_circ_0006117 sequence). Firefly luciferase (Luc) intensity was normalized to that of Renilla luciferase (Rluc). (i) A schematic diagram showing the putative conjugated site between miR-96-5p and the 3′UTR of *KRAS*. (j) The relative luciferase intensity in PC cells cotransfected with the miR-96-5p control or miR-96-5p mimics and KRAS-WT (containing the wild-type (WT) miR-96-5p-binding sequence) or KRAS-MUT (containing a mutated (MUT) miR-96-5p-binding sequence). Luc intensity was normalized to that of Rluc. All values were shown as means ± SD, ^*∗*^*P* < 0.05, and ^*∗∗*^*P* < 0.001.

The above studies have shown that circular RNA hsa_circ_0006117 may play PC-promoting effects by sponging miRNAs. Hence, we used bioinformatic analysis to explore which miRNAs can bind both circular RNA hsa_circ_0006117 and *KRAS*, and miR-96-5p was identified as a possible candidate (Figure 5(a)). RT-qPCR indicated that miR-96-5p was lower expressed in PC cells than in HPDE cells (Figure 5(b)). On the contrary, miR-96-5p content in PC cells transfected with circRNA-sh#1 and circRNA-sh#2 increased (Figure 5(c)), whereas the content of *KRAS* in PC cells transfected with miR-96-5p mimics reduced (Figures 5(d), and 5(e)). Furthermore, the data presented in Figure 5(f) showed that miR-96-5p content was inversely associated with the content of both circular RNA hsa_circ_0006117 and *KRAS*. These results suggested that circular RNA hsa_circ_0006117 and *KRAS* may share miR-96-5p binding sites. We subsequently generated pmiR-RB-Report™ constructs containing either circular RNA hsa_circ_0006117 or *KRAS* 3′UTR sequences and confirmed them by sequencing (Supplementary figures S4A–S4D). As expected, the luciferase intensity of PC cells cotransfected with miR-96-5p mimics and circRNA-WT was significantly weaker than that cotransfected with miR-96-5p mimics and circRNA-MUT, prompting that miR-96-5p and circular RNA hsa_circ_0006117 have complementary binding sequences (Figures 5(g) and 5(h)). Meanwhile, the same complementary sequences were also present in the *KRAS* 3′UTR (the predicted binding sites are displayed in Figure 5(i)). Subsequently, the results demonstrated that PC cells cotransfected with the miR-96-5p mimics KRAS-WT, but not KRAS-MUT, displayed reduced luciferase activity, suggesting that miR-96-5p interacted with *KRAS* by binding to sequences in its 3′UTR (Figure 5(j)).

### 3.6. Circular RNA hsa_circ_0006117 Facilitated PC Development by Adsorbing miR-96-5p

**Figure 6:**
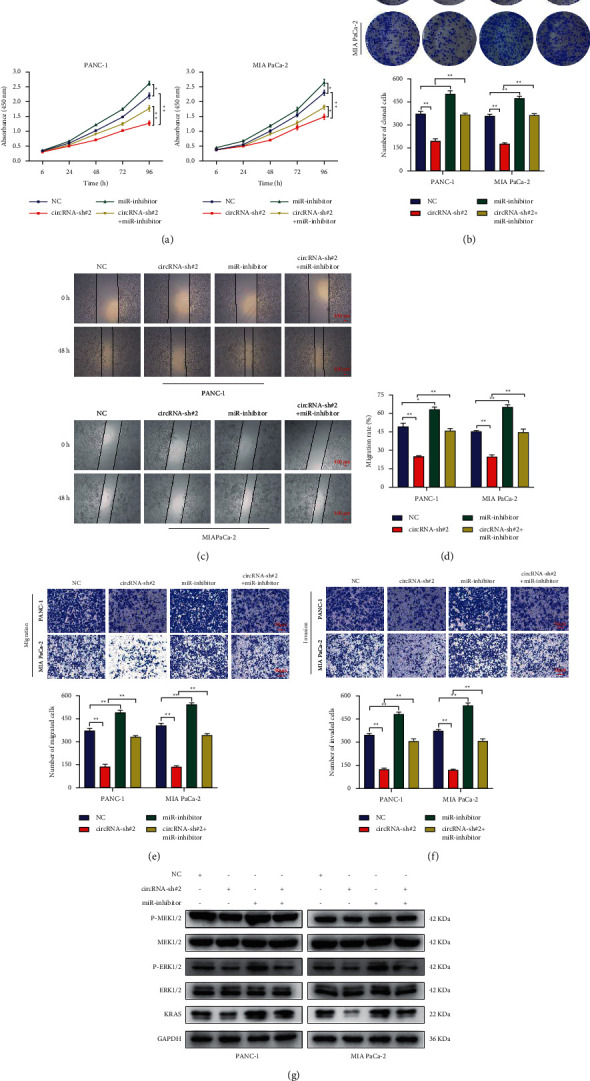
Circular RNA hsa_circ_0006117 facilitates pancreatic cancer (PC) development by adsorbing miR-96-5p. (a) The growth ability in circular RNA hsa_circ_0006117-silenced and (or) miR-96-5p-knockdown PC cells. (b) The colony-forming ability of PC cells in different processing groups. (c, d) The migratory ability of PC cells in different processing groups was verified by a wound healing assay. Representative images were obtained at ×40 magnification (bars: 100 *μ*m). (e, f) The invasive and migratory capabilities of PC cells were investigated by transwell migration (e) and invasion (f) assay, respectively. Representative images were obtained at ×200 magnification (bars: 50 *μ*m). (g) Protein expression of key genes in the MAPK signaling pathway in circular RNA hsa_circ_0006117-silenced and (or) miR-96-5p-knockdown PC cells. All values were shown as means ± SD, ^*∗*^*P* < 0.05, and ^*∗∗*^*P* < 0.001.

To investigate whether circular RNA hsa_circ_0006117 facilitates PC development by regulating miR-96-5p, in circular RNA hsa_circ_0006117-silenced PC cells, miR-96-5p-inhibitor was transfected or cotransfected for subsequent rescue experiments. Experiments reflecting proliferative capacity (Figures 6(a) and 6(b)) suggested that miR-96-5p-inhibitor facilitated the growth of PC cells, whereas cotransfection with miR-96-5p-inhibitor and circRNA-sh#2 prevented the inhibition of circular RNA hsa_circ_0006117, thereby accelerating the growth of PC cells. Similarly, following rescue experiments, wound healing (Figures 6(c) and 6(d)) and transwell (Figures 6(e) and 6(f)) assays demonstrated that cotransfection with miR-96-5p-inhibitor could restore the loss of circular RNA hsa_circ_0006117 expression and promote PC migration and invasion. Moreover, in PC cells where circular RNA hsa_circ_0006117 was knocked down, miR-96-5p-inhibitor rescued the phosphorylation of MEK1/2 and ERK1/2, which were key activators of the MAPK signaling pathway (Figure 6(g)).

## 4. Discussion

PC displays highly invasive and metastatic characteristics [23, 24]. Advances in the comprehensive systemic treatment of PC have not resulted in improvements in the prognosis of this cancer [25]. Numerous pieces of evidence have suggested that circRNAs have inspired the development of PC. A study by Li et al. [26] revealed that circ-IARS secreted by PC cells affected endothelial monolayer permeability, thereby facilitating PC invasion and metastasis. In addition, another study [14] suggested that circ-PDE8A could facilitate the invasiveness capability of PC cells through the stimulation of the MET/ERK or AKT pathways, and exosomal circ-PDE8A has been related to the PC process and the prognosis of PC patients. In contrast, reduced circRNA expression has also been linked with the inhibition of PC progression. For example, Kong et al. [6] revealed that circNFIB1 regulates the miR-486-5p/PIK3R1 axis and further suppresses lymphatic metastasis in PC. Analogously, circular RNA hsa_circ_0006117 is reported to be low expressed and identified as a tumor-inhibiting factor in non-small cell lung carcinomas [27] and bladder cancer [28] recently. Surprisingly, our analysis results suggested that circular RNA hsa_circ_0006117 was highly expressed in PC, which indicated that it may have tissue specific and play a cancer-promoting role in PC. After all, the tissue specificity of circRNAs is one of their most striking features. However, current studies have not found a cancer-promoting role of circular RNA hsa_circ_0006117. We reported for the first time that circular RNA hsa_circ_0006117 is upregulated and played a promotive role in PC. These clues provided novel insights for further exploring the role of circular RNA hsa_circ_0006117. Furthermore, our results confirmed the PC tissue specificity of circular RNA hsa_circ_0006117 expression and revealed its independent biological function.

The KRAS/MAPK signaling pathway is strongly related to the growth and survival of cancer cells [29, 30]. This pathway is also involved in resistance to chemotherapy, autophagy, and metabolic reprogramming, all of which contribute to the malignant capacity of PC [19, 31]. In addition, *KRAS* promotes PC development through the regulation of noncoding RNA or nucleotide synthesis, while *KRAS* is a well-known activator that mediates the phosphorylation/activation of the MAPK signaling pathway [32, 33]. Here, our results also support this idea. However, whether there is an association between *KRAS* and circRNAs in PC has not been investigated. In our research, we first demonstrated that circular RNA hsa_circ_0006117 could upregulate the expression of *KRAS via* the competitive absorption of miRNA. And we found the cancer-promoting role of miR-96-5p in PC, which is consistent with previous work by others [34]. Intriguingly, previous studies [17, 35] have identified *KRAS* as a candidate for genetic therapy for PC treatment. Further, a recently published study suggested that an inhibitor targeting mutated *KRAS* represented an effective treatment for some types of tumors [36]. Besides, therapies targeting MEK/ERK are considered to be promising treatments to slow the progression of PC [31]. Our study indicated that circular RNA hsa_circ_0006117 could stimulate the MAPK signaling pathway *via* up-regulating the phosphorylation of MEK/ERK and accelerate PC progression in a *KRAS*-dependent manner. Combined, we believed that circular RNA hsa_circ_0006117 may be a promising pharmacological target, and exploring the functions of *KRAS*-associated circRNAs may be of value for clinical application.

Accumulating evidence has indicated that circRNAs can play both oncogenic and tumor-suppressor roles *via* sponging miRNAs [37, 38]. For instance, Luo et al. [39] uncovered that circCCDC9 modulates the miR-6792-3p/CAV1 axis, thereby suppressing the development of gastric cancer. Another study revealed [40] that hsa_circ_001783 adsorbed miR-200c-3p to accelerate the malignant behavior of breast cancer. In our study, we also discovered that circular RNA hsa_circ_0006117 plays an oncogenic role in PC by sponging a miRNA (miR-96-5p). Notably, in similar research, most studies first identify miRNAs and then explore the associated downstream circRNA regulatory network. However, we first focused on the highly expressed genes in potential signaling pathways of PC and then searched for downstream targets and further determined the circRNA-related regulatory network, which led to miR-96-5p/*KRAS* and was identified as the downstream target of circular RNA hsa_circ_0006117 in PC. This provides a novel idea for the investigation of the ceRNA mechanism. Of course, it is undeniable that not all ceRNA mechanisms can be successfully verified by this approach. Combined, all of our work indicated that circular RNA hsa_circ_0006117 promotes PC progression in a manner that is dependent on the downregulation of miR-96-5p.

## 5. Conclusion

**Figure 7:**
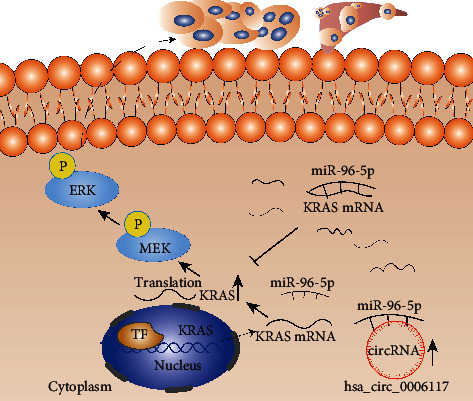
Schematic diagram of this research. Circular RNA hsa_circ_0006117 facilitates the malignant features of PC by regulating the miR-96-5p/KRAS/MAPK signaling pathway.

In summary, we have identified circular RNA hsa_circ_0006117 as a specifically highly expressed circRNA in PC. We further found that circular RNA hsa_circ_0006117 facilitates the malignant behaviors of PC through regulating the miR-96-5p/KRAS/MAPK signaling pathway (Figure 7). These results suggest that circular RNA hsa_circ_0006117 may contribute to the potential therapeutic target of PC.

## Figures and Tables

**Table 1:** Sequences of primers and oligonucleotides.

Gene		Sequence (5′->3′)
Circular RNA hsa_circ_0006117	Forward primer	CCAGATAACCAGTTCACGGATG
	Reverse primer	GGAATCCATGCTTATCTGAAGG
KRAS	Forward primer	GCAAGTAGTAATTGATGGAGAAACC
	Reverse primer	GCAAATACACAAAGAAAGCCCT
PTPRA	Forward primer	GCACCAACAGGAAATACCCA
	Reverse primer	GAAGAGCTTATTGTCGTCTGCC
GAPDH	Forward primer	GAACGGGAAGCTCACTGG
	Reverse primer	GCCTGCTTCACCACCTTCT
GRB2	Forward primer	CTGGGTGGTGAAGTTCAATTCT
	Reverse primer	GTTCTATGTCCCGCAGGAATATC
IGF2BP2	Forward primer	AGCTAAGCGGGCATCAGTTTG
	Reverse primer	CCGCAGCGGGAAATCAATCT
RAP1A	Forward primer	CGTGAGTACAAGCTAGTGGTCC
	Reverse primer	CCAGGATTTCGAGCATACACTG
*Oligonucleotides*		
miR-96-5p mimics	Sense strand	UUUGGCACUAGCACAUUUUUGCU
	Antisense strand	AGCAAAAAUGUGCUAGUGCCAAA
si-hsa_circ_0006117#1	CTTCAGATAAGCATGGATT	
si-hsa_circ_0006117#2	TCCTTCAGATAAGCATGGA	
si-hsa_circ_0006117#3	TCCCTCCTTCAGATAAGCA	

## Data Availability

The data used to support the findings of this study are included within the article and supplementary information files.

## Supplementary Materials

Supplementary Materials Supplementary Figure S1: (A-B) the heat map obtained by R analysis showed the differentially expressed circRNAs in GSE69362 and GSE79634 datasets, respectively; (C) the relative mRNA content of circular RNA hsa_circ_0006117 and PTPRA in PC cells after actinomycin D treatment. All values were expressed as means ± SD, ns: not significant, ^*∗∗*^*P* < 0.001. Supplementary Figure S2: the pattern diagram of circular RNA hsa_circ_0006117 small interfering RNA (siRNA). Supplementary Figure S3: (A) potential target genes (red) enriched on the MAPK signaling pathway; (B) potential target genes (red) enriched on the RAS signaling pathway; (C) differently expressed GRB2, IGF2BP2, and RAP1A from the GEPIA database; (D) the mRNA content of GRB2, IGF2BP2, and RAP1A in PC cells transfected with shRNA of circular RNA hsa_circ_0006117. All values were expressed as means ± SD, ^*∗*^*P* < 0.05. Supplementary Figure S4: (A-B) sequencing results of pmiR-RB-Report™ circular RNA hsa_circ_0006117-WT (A) and pmiR-RB-Report™ circular RNA hsa_circ_0006117-MUT (B); (C-D) sequencing results of pmiR-RB-Report™ KRAS-WT (C) and pmiR-RB-Report™ KRAS-MUT (D).
