# Supplementary material for: Circular RNA hsa_circ_0006117 Facilitates Pancreatic Cancer Progression by Regulating the miR-96-5p/KRAS/MAPK Signaling Pathway
Source: J Oncol. 2021 Sep 2;2021:9213205. doi: 10.1155/2021/9213205 (PMC8433013; doi:10.1155/2021/9213205)
Supplement: Supplementary Materials — Supplementary Figure S1: (A-B) the heat map obtained by R analysis showed the differentially expressed circRNAs in GSE69362 and GSE79634 datasets, respectively; (C) the relative mRNA content of circular RNA hsa_circ_0006117 and PTPRA in PC cells after actinomycin D treatment. All values were expressed as means ± SD, ns: not significant, ∗∗P < 0.001. Supplementary Figure S2: the pattern diagram of circular RNA hsa_circ_0006117 small interfering RNA (siRNA). Supplementary Figure S3: (A) potential target genes (red) enriched on the MAPK signaling pathway; (B) potential target genes (red) enriched on the RAS signaling pathway; (C) differently expressed GRB2, IGF2BP2, and RAP1A from the GEPIA database; (D) the mRNA content of GRB2, IGF2BP2, and RAP1A in PC cells transfected with shRNA of circular RNA hsa_circ_0006117. All values were expressed as means ± SD, ∗P < 0.05. Supplementary Figure S4: (A-B) sequencing results of pmiR-RB-Report™ circular RNA hsa_circ_0006117-WT (A) and pmiR-RB-Report™ circular RNA hsa_circ_0006117-MUT (B); (C-D) sequencing results of pmiR-RB-Report™ KRAS-WT (C) and pmiR-RB-Report™ KRAS-MUT (D). [file 9213205.f1.docx]

**Supplementary Materials**


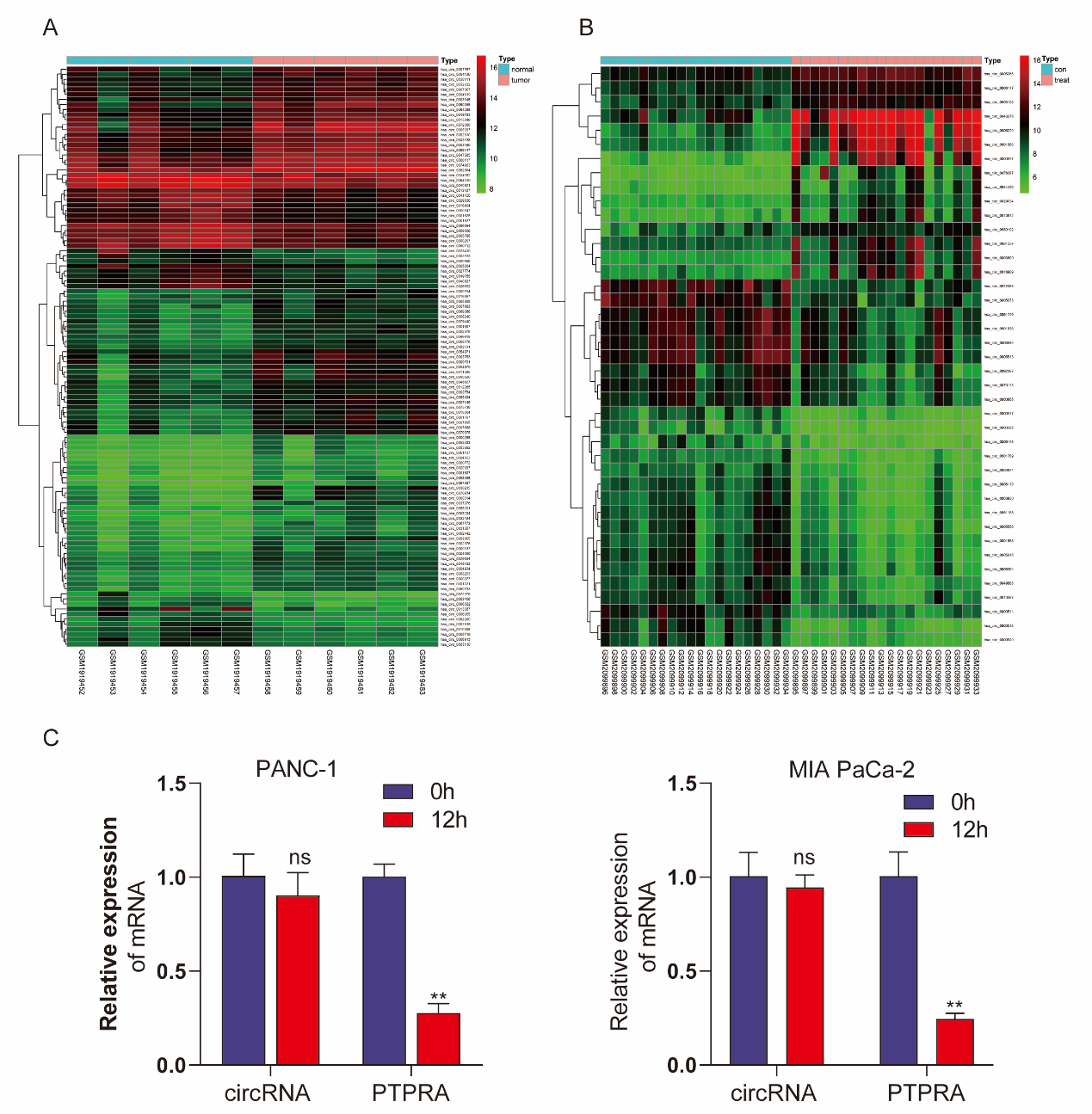


**Supplementary** **figure S1： (A-B)** The heat map obtained by R analysis showed the differentially expressed circRNAs in GSE69362 and GSE79634 datasets, respectively. (**C)** The relative mRNA content of circular RNA hsa_circ_0006117 and PTPRA in PC cells after Actinomycin D treatment. All values were shown as means ± SD, ns: not significant, ***P* < 0.001.


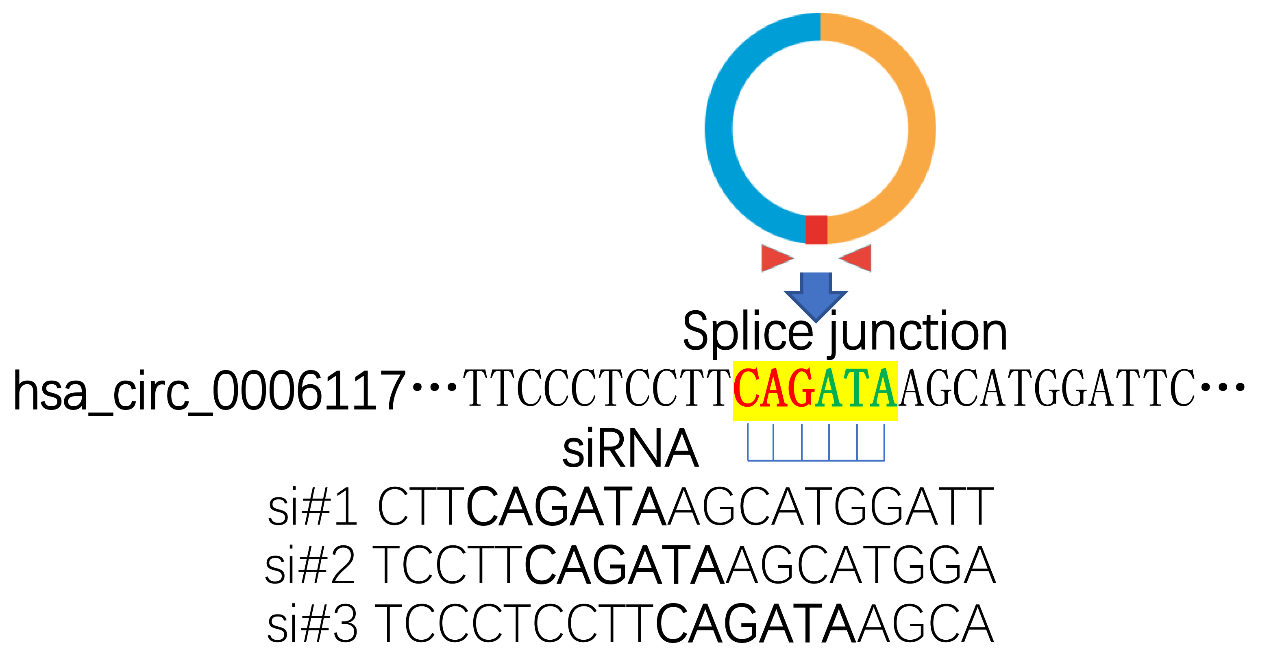


**Supplementary** **figure S2：** The pattern diagram of circular RNA hsa_circ_0006117 small interfering RNA (siRNA).


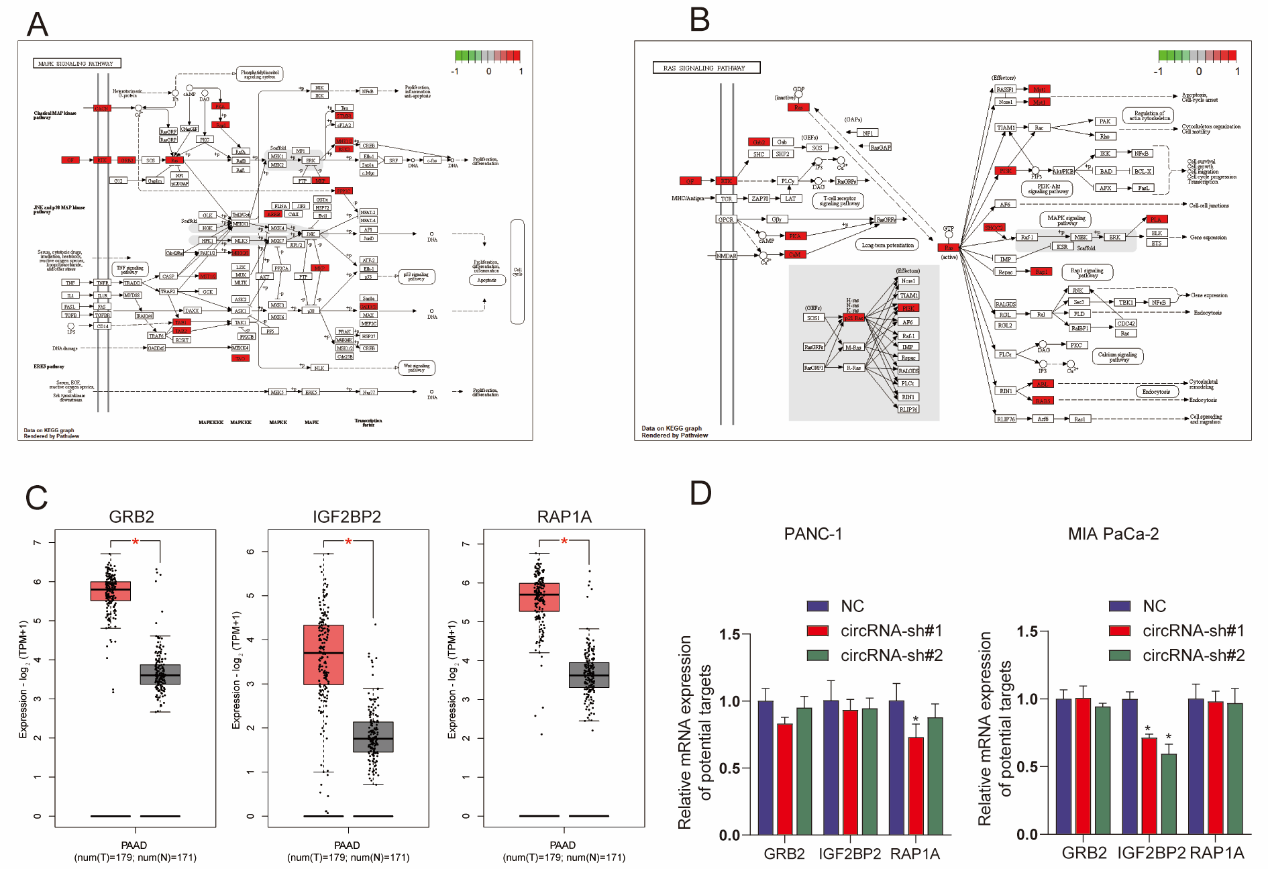


**Supplementary figure S3： (A)** Potential target genes (red) enriched on the MAPK signaling pathway. **(B)** Potential target genes (red) enriched on the RAS signaling pathway. **(C)** Differently expressed GRB2, IGF2BP2, and RAP1A from the Gene Expression Profiling Interactive Analysis (GEPIA) database (http://gepia2.cancer-pku.cn/#index). **(D)** The mRNA content of GRB2, IGF2BP2, and RAP1A in PC cells transfected with shRNA of circular RNA hsa_circ_0006117. All values were shown as means ± SD, **P* < 0.05.


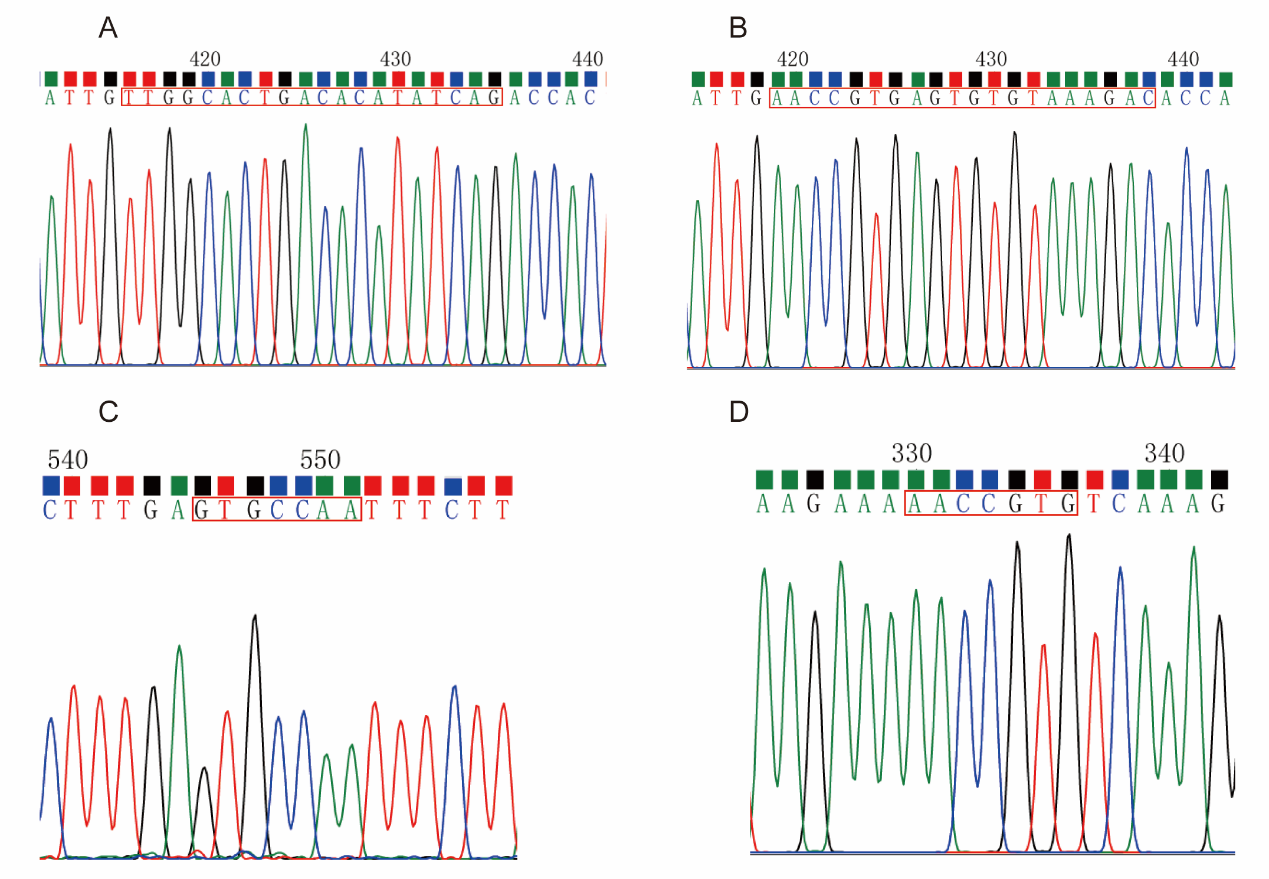


**Supplementary** **figure S4： (A-B)** Sequencing results of pmiR-RB-Report^TM^ circular RNA hsa_circ_0006117-WT **(A)** and pmiR-RB-Report^TM^ circular RNA hsa_circ_0006117-MUT **(B)**. **(C-D)** Sequencing results of pmiR-RB-Report^TM^ KRAS-WT **(C)** and pmiR-RB-Report^TM^ KRAS-MUT **(D)**.
